# Generation and Analysis of Novel Plant-Derived Antibody-Based Therapeutic Molecules against West Nile Virus

**DOI:** 10.1371/journal.pone.0093541

**Published:** 2014-03-27

**Authors:** Junyun He, Huafang Lai, Michael Engle, Sergey Gorlatov, Clemens Gruber, Herta Steinkellner, Michael S. Diamond, Qiang Chen

**Affiliations:** 1 The Biodesign Institute and School of Life Sciences, Arizona State University, Tempe, Arizona, United States of America; 2 College of Technology and Innovation, Arizona State University, Mesa, Arizona, United States of America; 3 Department of Medicine, Washington University School of Medicine, St Louis, Missouri, United States of America; 4 Department of Molecular Microbiology, Washington University School of Medicine, St Louis, Missouri, United States of America; 5 Department of Pathology & Immunology, Washington University School of Medicine, St Louis, Missouri, United States of America; 6 Department of Chemistry, University of Natural Resources and Applied Life Sciences, Vienna, Austria; 7 Department of Applied Genetics and Cell Biology, University of Natural Resources and Applied Life Sciences, Vienna, Austria; 8 MacroGenics, Inc, Rockville, Maryland, United States of America; University of Texas Medical Branch, United States of America

## Abstract

Previously, our group engineered a plant-derived monoclonal antibody (MAb) (pHu-E16) that efficiently treated West Nile virus (WNV) infection in mice. In this study, we developed several pHu-E16 variants to improve its efficacy. These variants included a single-chain variable fragment (scFv) of pHu-E16 fused to the heavy chain (HC) constant domains (C_H_^1-3^) of human IgG (pHu-E16scFv-C_H_^1-3^) and a tetravalent molecule (Tetra pHu-E16) assembled from pHu-E16scFv-C_H_^1-3^ with a second pHu-E16scFv fused to the light chain (LC) constant region. pHu-E16scFv-C_H_^1-3^ and Tetra pHu-E16 were efficiently expressed and assembled in plants. To assess the impact of differences in N-linked glycosylation on pHu-E16 variant assembly and function, we expressed additional pHu-E16 variants with various combinations of HC and LC components. Our study revealed that proper pairing of HC and LC was essential for the complete N-glycan processing of antibodies in both plant and animal cells. Associated with their distinct N-glycoforms, pHu-E16, pHu-E16scFv-C_H_^1-3^ and Tetra pHu-E16 exhibited differential binding to C1q and specific Fcγ receptors (FcγR). Notably, none of the plant-derived Hu-E16 variants showed antibody-dependent enhancement (ADE) activity in CD32A^+^ human cells, suggesting the potential of plant-produced antibodies to minimize the adverse effect of ADE. Importantly, all plant-derived MAb variants exhibited at least equivalent *in vitro* neutralization and *in vivo* protection in mice compared to mammalian cell-produced Hu-E16. This study demonstrates the capacity of plants to express and assemble a large, complex and functional IgG-like tetravalent mAb variant and also provides insight into the relationship between MAb N-glycosylation, FcγR and C1q binding, and ADE. These new insights may allow the development of safer and cost effective MAb-based therapeutics for flaviviruses, and possibly other pathogens.

## Introduction

The development and implementation of targeted monoclonal antibody (MAb) therapy have provided new opportunities for controlling a wide range of diseases. Although MAbs produced in mammalian cell culture systems have achieved remarkable clinical success, their high cost, long manufacturing time, and restricted production capacity have limited the availability, utility and potential of these drugs. Several of these challenges might be overcome by using plant expression systems, because they offer scalable production of MAbs at low cost with a low risk of introducing adventitious human or animal pathogens [1]–[3]. Functional antibody production requires a eukaryotic host cell that can assemble four antibody polypeptides into a heterotetramer and perform complex N-linked glycosylation. Despite this complexity, a MAb was successfully expressed in tobacco plants only three years after the first plant-made biologic [4]. Since then, a variety of MAbs and their derivatives, such as IgG, IgA, single-chain variable fragments (scFv), and diabodies have been produced in plants [3]. The largest reported MAb-based molecule produced in plants is a recombinant immune complex (RIC) [5]. The ability of plants to express and assemble larger or more complex MAb-derived molecules such as tetravalent MAbs or bifunctional MAbs has not been described.

N-linked glycosylation of proteins occurs as a series of post-translational modification steps in host cells and depends on the proper folding of the target protein and its transport to the appropriate endomembrane compartments [6]. As such, MAb variants with significant polypeptide structural differences from the native molecule also may have appreciable differences in glycan structures. Structural differences also may impact the pharmacokinetics, antigen binding, stability, effector functions, immunogenicity, and efficacy of a MAb and its derivatives.

West Nile virus (WNV) is a neurotropic virus that infects the central nervous system (CNS) of human and animals. Historically, WNV was an Old World disease found mostly in the Eastern Europe, Africa, and the Middle East. However, in 1999, WNV entered the Western hemisphere and subsequently spread across the United States (US), Canada, the Caribbean region and Latin America [7] with outbreaks occurring on an annual basis. The elderly and immunocompromised are the most vulnerable for developing severe neurological disease, long-term morbidity, and death [8], although genetic factors also are associated with an increase risk of disease [9], [10]. Currently, there is no vaccine or therapeutic approved for human use. The global threat of WNV epidemics and the lack of treatment warrant the development of antiviral therapeutics and production platforms that can bring products to market at low cost. We previously reported a plant-derived, humanized murine MAb (pHu-E16) that binds to an epitope on domain III (DIII) of WNV envelope (E) protein, as a post-exposure therapeutic candidate for WNV [11]. We demonstrated that pHu-E16 was produced at high levels and assembled efficiently in both *Nicotiana benthamiana* and lettuce plants [11], [12]. pHu-E16 retained antigen binding specificity, neutralized WNV infection, and protected mice from lethal infection equivalently compared to the mammalian cell-produced Hu-E16 (mHu-E16) [11]. Because WNV is a neurotropic virus, peripheral delivery of pHu-E16, however, likely will have a limited window of efficacy due to its inability to cross the blood brain barrier (BBB) efficiently and accumulate in the brain at concentrations sufficient for neutralization. Thus, it would be desirable to develop pHu-E16 variants, such as bifunctional MAbs, that can cross the BBB while retaining targeted therapeutic activity.

To test the ability of plants in producing such complex MAb variants, here we expressed several pHu-E16 derivatives including a pHu-E16scFv fused to the heavy chain (HC) constant domains (C_H_^1-3^) of human IgG (pHu-E16scFv-C_H_^1-3^) and a large tetravalent molecule (Tetra pHu-E16) assembled from pHu-E16scFv-C_H_^1-3^ with another pHu-E16scFv fused to the light chain (LC) constant region (C_L_) (pHu-E16scFv-C_L_). We demonstrated that plants can express and assemble these pHu-E16 variants efficiently. These pHu-E16 variants also showed at least equivalent protection as the parent pHu-E16 or mHu-E16 against a lethal WNV challenge in a mouse model. Our results also revealed differences in N-linked glycosylation pattern between different MAb variants, and demonstrated that the proper pairing of HC and LC was essential for the complete N-glycan processing of antibodies in both plant and animal cells. We also found that pHu-E16, pHu-E16scFv-C_H_^1-3^ and Tetra pHu-E16 exhibited differential binding to specific Fcγ receptors (FcγRs) and C1q, the complement opsonin that activates the classical complement pathway. In human K562 cells, none of the plant-derived Hu-E16 variants showed significant antibody-dependent enhancement (ADE) activity, a phenomenon that is an impediment for developing MAb-based therapeutics against flaviviruses, such as the related Dengue virus. Overall, this study demonstrates the capacity of a plant to assemble a large and complex IgG-like tetravalent MAb, which offers a step forward toward the development of bifunctional MAbs in plants.

## Results

### Expression and assembly of pHu-E16 scFv-C_H_^1-3^ in *N. benthamiana*

**Figure 1:**
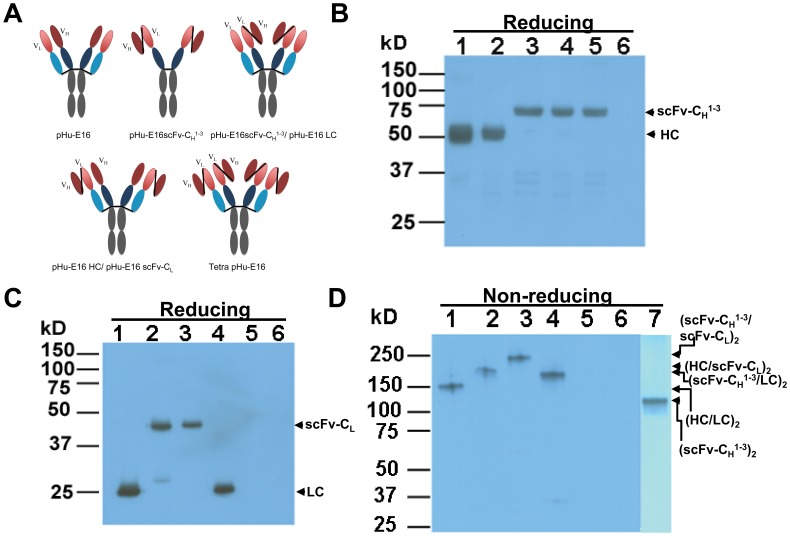
Western blot analysis of pHu-E16 variants. **A**. Designs of Hu-E16 variants in this study. **B–D**. Western blot analysis. Hu-E16 variants were extracted from *N. benthamiana* leaves, separated on SDS-PAGE gels under reducing (**B** and **C**) or non-reducing (**D**) conditions, and blotted onto PVDF membranes. The membranes were incubated with a goat anti-human gamma chain antibody or a goat anti-human kappa chain antibody to detect heavy chain (**B** and **Lane 7 of D**) or light chain (**C** and **Lanes 1–6 of D**). Lane 1, pHu-E16 as a reference standard; lane 2, Protein sample extracted from leaves co-infiltrated with pHu-E16 HC and pHu-E16scFv-C_L_ constructs; lane 3, Sample from leaves co-infiltrated with pHu-E16scFv-C_H_^1-3^ and pHu-E16scFv-C_L_; lane 4, Sample from leaves co-infiltrated with pHu-E16scFv-C_H_^1-3^ and pHu-E16 LC; lanes 5 and 7, Sample from leaves infiltrated with pHu-E16scFv-C_H_^1-3^; lane 6, Sample from un-infiltrated leaves. HC: heavy chain, scFv: single-chain variable fragment; C_H_^1-3^: the constant region domains 1 to 3 of HC; LC: light chain; C_L_: Constant region of LC; V_L_: variable region of LC; V_H_: variable region of HC.

**Figure 2:**
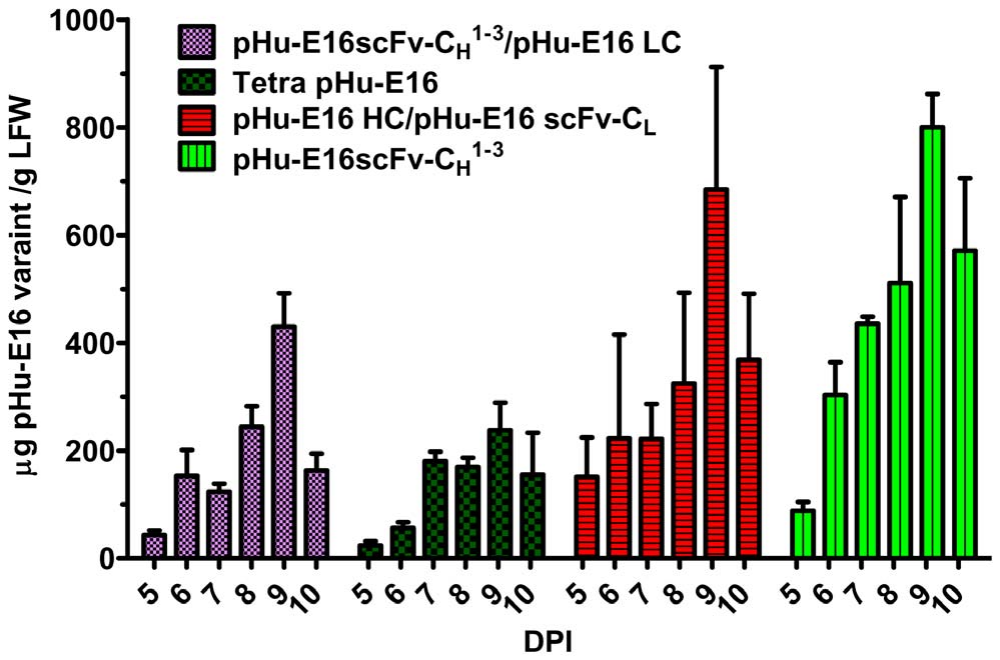
Time course of pHu-E16 expression in *N. benthamiana* plants. Total proteins from leaves infiltrated or co-infiltrated with pHu-E16scFv-C_H_^1-3^, pHu-E16 HC/pHu-E16scFv-C_L_, pHu-E16scFv-C_H_^1-3^/pHu-E16scFv-C_L_ (Tetra pHu-E16), or pHu-E16scFv-C_H_^1-3^/pHu-E16 LC were extracted on days 5 -10 post infiltration and analyzed by an ELISA that detects the assembled form of pHu-E16 MAb variants, Mean ± SD of samples from three independent infiltrations are presented.

An important prerequisite for the generation of bifunctional Hu-E16 MAb is the correct assembly of its functional component: pHu-E16scFv-C_H_^1-3^. The DNA sequence of the variable region of HC (V_H_) and LC (V_L_) of pHu-E16 [11] was fused together first to generate pHu-E16scFv, and then fused to the coding sequence of the C_H_^1-3^ of human IgG1 (**Fig 1A**). The resulting coding sequence of pHu-E16scFv-C_H_^1-3^ was cloned into a MagnICON-based plant expression vector [13], and transformed into *Agrobacterium tumefaciens*. *A. tumefaciens* strains containing the pHu-E16scFv-C_H_^1-3^ construct were co-delivered into *N. benthamiana* leaves along with the promoter module and an integrase construct through agroinfiltration [14], [15]. Western blot analysis after reducing or non-reducing gel electrophoresis confirmed that pHu-E16scFv-C_H_^1-3^ was produced in leaves with the expected molecular weight (**Fig 1B** **, Lane 5**), and that it assembled into a dimer (**Fig 1D** **, Lane 7**). ELISA results also indicated that pHu-E16scFv-CH^1-3^ reached the highest level of accumulation 9 days post infiltration (dpi) with *A. tumefaciens*, with an average accumulation of 0.8 mg/g leaf fresh weight (LFW) (**Fig 2**). This level is similar to that previously reported for the parent pHu-E16 MAb, and is among the highest expression levels for MAbs in plants ever described [3], [11], [16].

### pHu-E16 scFv-C_H_^1-3^ exhibited altered N-linked glycosylation pattern

A typical feature of IgG antibodies is a conserved N-linked glycosylation site at position Asn^297^ in the C_H_^2^ domain; it is well documented that the pattern and extent of N-linked glycosylation affects the stability and function of an antibody [17]. Accordingly, we next examined the N-glycoforms of pHu-E16scFv-C_H_^1-3^ by liquid-chromatography-electrospray ionization-mass spectrometry (LC-ESI-MS) to assess whether the extent of N-linked glycosylation was similar to that of the parent pHu-E16. pHu-E16 exhibited the expected complex-type N-glycans (GnGnXF, **Table 1**) that are typical for plant-produced IgGs [18]. However, pHu-E16scFv-C_H_^1-3^ displayed oligomannosidic structures, predominantly Man8 and Man9 (**Table 1**). The predominantly endoplasmic reticulum (ER)-type N-glycans of pHu-E16scFv-C_H_^1-3^ were unexpected, as this molecule was targeted for secretion and not tagged with an ER-retention signal KDEL. To investigate if this is a phenomenon specific to plant cells, we also compared the N-glycan profiles of the same Hu-E16 variants produced in mammalian cells (mHu-E16 and mHu-E16scFv-C_H_^1-3^). We observed a substantial amount of oligomannosidic glycans (oligoman, mostly M5 and M7) in mHu-E16scFv-C_H_^1-3^ in addition to the predominant complex-type glycans (GnGnF^6^ and AGnF^6^), whereas no such glycans were observed in mHu-E16 (**Table 1**). This result suggests that the unusual N-linked glycosylation pattern of scFv-C_H_^1-3^ is not unique to plants but also is shared by mammalian cells, even though the percentage and the particular species of oligomannosidic glycans differ between cells from the two species.

**Table 1:** Relative abundance in percentage of major glyco-structures detected on Hu-E16 variants.

	A	B	C	D	E	F	G
**Glycan Structure**	pHu-E16	pHu-E16scFv-C_H_^1-3^	pHu-E16scFv-C_H_^1-3^/ LC	HC/ pHu-E16scFv-C_L_	pHu-E16scFv-C_H_^1-3^/pHu-E16 scFv-C_L_	mHu-E16	mHu-E16scFv-C_H_^1-3^
**GnGnXF**	90		27	88	19		
**∑ oligoman**		90	66	8	72		10
**GnGnF6**						78	65
**AGnF^6^**						15	20
**∑ other**	10	10	7		9	8	5

∑other complex: sum of glycoforms present at levels below 5%. The glycan structures are assigned using the ProGlycAn nomenclature (www.proglycan.com). HC: heavy chain, C_H_^1-3^: the constant regions 1–3 of HC, LC: light chain, C_L_: constant region of LC.

### Expression and N-linked glycosylation of a tetravalent pHu-E16 and other pHu-E16 variants

The success of pHu-E16 scFv-C_H_^1-3^ expression and assembly in plants indicates the feasibility of developing an IgG-like tetravalent MAb variant. Such a form could provide four WNV antigen-binding sites or enable development of bifunctional MAbs that offer divalent binding to two different antigens. For example, bifunctional MAbs could be engineered to bind a BBB receptor as its second antigen to enhance transport into the brain and extend the window of treatment for neuroinvasive WNV infection. To understand the basis of the difference in N-linked glycans between pHu-E16scFv-C_H_^1-3^ and its parent pHu-E16 and develop a potentially more effective WNV therapeutic, we engineered and expressed three additional pHu-E16 variants with various combinations of HC and LC components. First, pHu-E16scFv was fused to the plant-codon optimized C_L_ (pHu-E16scFv-C_L_). Three pHu-E16 variants were then produced in *N. benthamiana* plants by the co-expression of the following Hu-E16 construct combinations: scFv-CH^1-3^/LC, HC/scFv-C_L_, and scFv-C_H_^1-3^/scFv-C_L_ (**Fig 1A**). Western blot analysis after reducing gel electrophoresis confirmed that each component of the variants was produced in plants with the expected molecular weight (**Fig 1B and C** **, Lanes 2–4**). These protein bands were specific to the infiltrated molecular constructs and were not detected in samples from negative control plant leaves (**Fig 1B and C** **, Lane 6**). Moreover, western blotting after non-reducing gel electrophoresis revealed that the two polypeptides in each combination assembled into an IgG-like heterotetramer (**Fig 1D** **, Lanes 2–4 and** **Fig 1A**). The successful expression and assembly of pHu-E16scFv-C_H_^1-3^ with pHu-E16scFv-C_L_ to form a tetravalent molecule (Tetra pHu-E16, **Fig 1B-D** **, Lane 3, and** **Fig 1A**) in plants is noteworthy, as the assembly of such large and complex MAb variants has not been reported previously. Yield analysis by ELISA established that Tetra pHu-E16 and other variants reached the highest level of production at 9 dpi. Nonetheless, the expression level varied between the three pHu-E16 variants, with HC/scFv-C_L_'s comparable to that of the parent pHu-E16 and pHu-E16scFv-C_H_^1-3^ (690 μg/g LFW), scFv-CH^1-3^/LC's (430 μg/g LFW), and Tetra pHu-E16 (240 μg/g LFW) produced at slightly lower levels (**Fig 2**).

In addition to developing a tetravalent pHu-E16 as a possible novel WNV therapeutic molecule, the expression of the three pHu-E16 variants also allowed us to study the cause of the unexpected N-linked glycosylation of pHu-E16scFv-C_H_^1-3^, specifically to address whether LC or the proper pairing of HC and LC affected N-linked glycosylation of MAb and its variants. Co-expression of LC of pHu-E16 increased the percentage of complex-type glycans in pHu-E16scFv-C_H_^1-3^ (**Table 1**). In Tetra pHu-E16, the co-expression and pairing of scFv-C_H_^1-3^/scFv-C_L_ also increased the amount of complex-type N-glycans (**Table 1**), albeit not to the same level as in the scFv-C_H_^1-3^/LC combination. In contrast, pairing of the HC of pHu-E16 with scFv-C_L_ yielded a predominantly complex-type N-linked glycosylation pattern, similar to that of the parent pHu-E16 (**Table 1**). These results indicated the importance of LC pairing with HC for the complete processing of N-linked glycans on antibodies.

### Purification of pHu-E16 variants from *N. benthamiana* leaves

**Figure 3:**
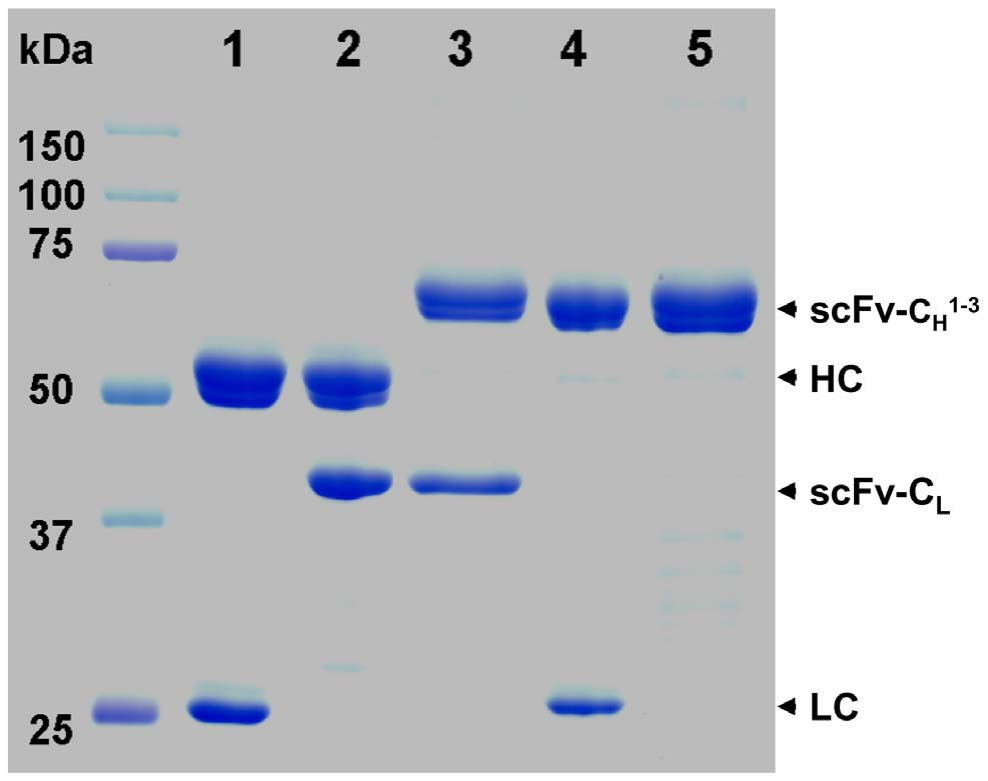
Purification of pHu-E16 variants from N. benthamiana leaves. Leaf proteins were extracted on day 9 after agroinfiltration. pHu-E16 variants were purified and analyzed on a 4-20% gradient SDS-PAGE gel under reducing condition and visualized with Coomassie stain. Lane 1, pHu-E16 as a reference standard; lane 2, pHu-E16 HC/pHu-E16scFv-C_L_; lane 3, Tetra pHu-E16; lane 4, pHu-E16scFv-C_H_^1-3^/pHu-E16 LC; lanes 5, pHu-E16scFv-C_H_^1-3^. Ten μg of purified protein was loaded in each lane of the gel. One representative of several independent experiments is shown.

For Tetra pHu-E16 and other variants to be feasible therapeutic candidates for WNV, an efficient extraction and purification process must be developed. We applied a scalable extraction and purification process previously developed for pHu-E16 to the purification of these variants [11]. Analysis of processing samples with gel electrophoresis indicate that in spite of their structural complexity, Tetra pHu-E16, pHu-E16scFv-C_H_^1-3^, and other variants were readily extracted from plant tissue and enriched to >90% purity by a combination of ammonium sulfate precipitation and protein A chromatography (**Fig 3**). These purified pHu-E16 variants were used for further functional studies.

### pHu-E16 variants show differential binding to human Fcγ receptors and C1q and reduced antibody-dependent enhancement activity

**Figure 4:**
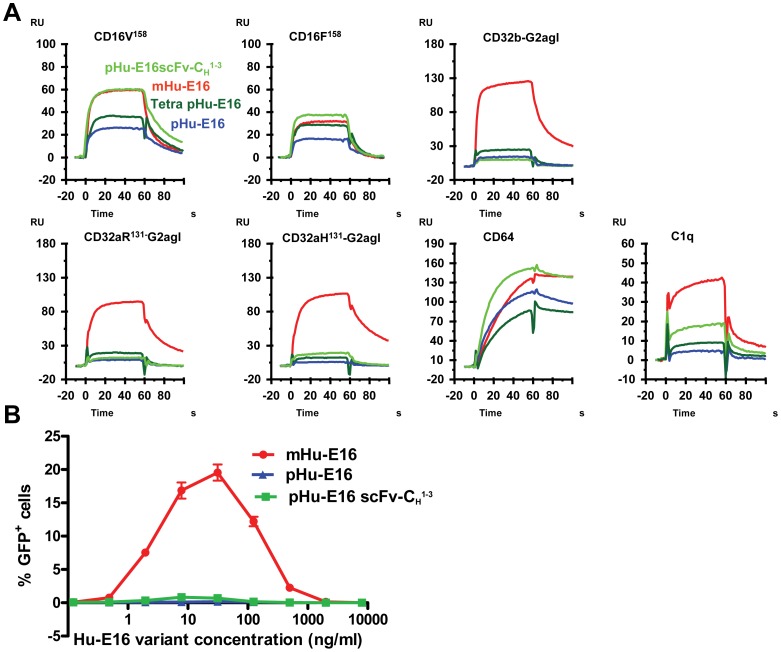
FcγR and C1q binding and antibody-dependent enhancement of pHu-E16 variants. **A**. SPR analysis of FcγR and C1q binding. FcγRs including CD16A (CD16V^158^, CD16F^158^), CD32A (CD32A-R^131^ and CD32A-H^131^ fused to an aglycosylated Fc region of IgG2 (CD32A-R^131^-G2agl and CD32A-H^131^-G2agl)), CD32B (CD32B fused to an aglycosylated Fc region of IgG2 (CD32B-G2agl)) and CD64, and C1q were injected at the same concentration over the surfaces with Hu-E16 variants captured on immobilized WNV E protein. Buffer injection was subtracted as blank and responses were normalized to same level of captured antibodies. **B**. ADE of WNV infection with antibody variants. Serial dilutions of Hu-E16 variants were mixed with WNV RVP and added to CD32A-expressing K562 cells. Forty-eight hours later, cells were analyzed by flow cytometry for GFP expression.

N-linked glycosylation in the Fc region of an antibody affects binding to FcγRs and C1q [17]. Accordingly, we investigated the binding of pHu-E16scFv-C_H_^1-3^ and Tetra pHu-E16 to C1q and different human FcγRs including CD16A-V^158^, CD16A-F^158^, CD32A-R^131^, CD32A-H^131^, CD32B, and CD64 by surface plasmon resonance (SPR) analysis using pHu-E16 and mHu-E16 as references. Compared to mHu-E16, pHu-E16 showed reduced binding to all FcγRs and C1q (**Fig 4A**). pHu-E16scFv-C_H_^1-3^ showed slightly enhanced binding to CD64 and the low-affinity (F^158^) isoform of CD16A, maintained similar binding to the high-affinity (V^158^) CD16A isoform, and had reduced binding to CD32A and B (**Fig 4A**). Tetra pHu-E16 showed a similar binding profile of FcγRs and C1q as pHu-E16scFv-CH^1-3^, except with reduced binding to CD64 and the high affinity isoform (V^158^) of CD16A (**Fig 4A**).

The interaction between Fc moieties and FcγRs or C1q also regulates antibody-dependent enhancement of infection (ADE), an *in vitro* phenomenon that relevant to disease pathogenesis of some flaviviruses [19]-[21]. As such, we investigated if plant-produced Hu-E16 variants would have a unique ADE profile *in vitro* compared to that of mHu-E16. As seen previously [22], mHu-E16, which has a native Fc moiety including a key N-linked glycan at position 297, efficiently promoted ADE in K562 cells that express the human FcγR CD32A (**Fig 4B**). In comparison, all pHu-E16 variants tested did not display ADE activity in human K562 cells, indicating the potential of plants to produce antibodies or antibody-like molecules that minimize ADE.

### Antigen binding and neutralization activity of pHu-E16 variants

**Figure 5:**
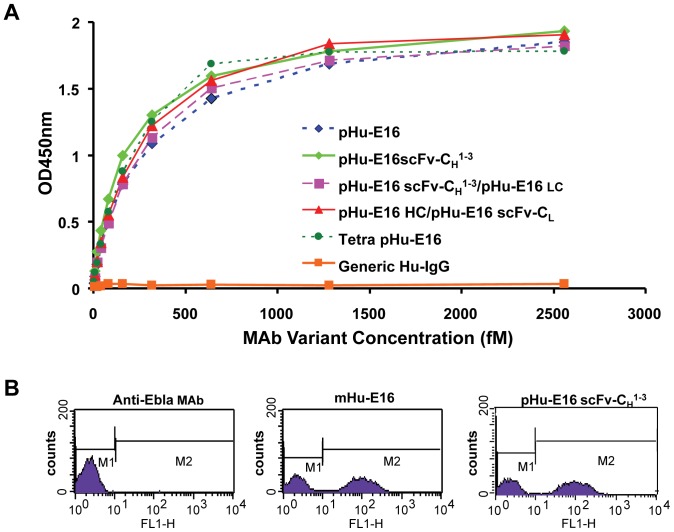
Antigen binding of pHu-E16 variants to DIII of WNV E. **A**. ELISA analysis. Serial dilutions of pHu-E16 variants were incubated on plates coated with WNV DIII and detected with a HRP-conjugated anti-human gamma chain antibody. Dilutions of pHu-E16 were used in parallel as reference standards. A commercial generic human IgG (Southern Biotech) was used as a negative control. One set of representative O.D. 450 nm readings from several independent experiments is presented. **B**. Binding of pHu-E16scFv-C_H_^1-3^ to domain III of WNV E displayed on the cell surface of yeast. Yeast cells displaying domain III of WNV E protein were stained with pHu-E16scFv-C_H_^1-3^, mHu-E16 (positive control), or a plant-produced humanized MAb against Ebola virus GP1 protein (negative control). Yeast cells were then processed by flow cytometry. Representative data from three independent experiments are shown.

We also assessed the relative binding and function of pHu-E16 variants in several assays. The binding of pHu-E16 variants to WNV E DIII was determined by ELISA in which DIII was coated on microtiter plates [11]. E16scFv-C_H_^1-3^, Tetra pHu-E16 and other pHu-E16 variants bound in a similar manner to DIII as pHu-E16 (**Fig 5A**). Recognition of pHu-E16 variants for DIII was corroborated in a binding assay with yeast that displayed DIII on their surface. Flow cytometric analysis showed that the percentage of positive yeast and the mean fluorescence intensity of binding by saturating concentrations of pHu-E16scFv-C_H_^1-3^ and mHu-E16 were similar (**Fig 5B**). Analogous results were obtained for Tetra pHu-E16 and other variants (data not shown). The binding of these variants to WNV E DIII was quantitated by a SPR assay with the antibody captured on the chip and the recombinant monomeric DIII flowed over in solution; as expected, E16scFv-C_H_^1-3^ and Tetra pHu-E16 had similar monovalent binding affinity and kinetics (10 to 25 nM) for WNV E protein and DIII compared to its parent mHu-E16 (data not shown). These results confirm that E16scFv-C_H_^1-3^ and Tetra pHu-E16 retained antigen-binding activity and were comparable to pHu-E16 and mHu-E16.

**Figure 6:**
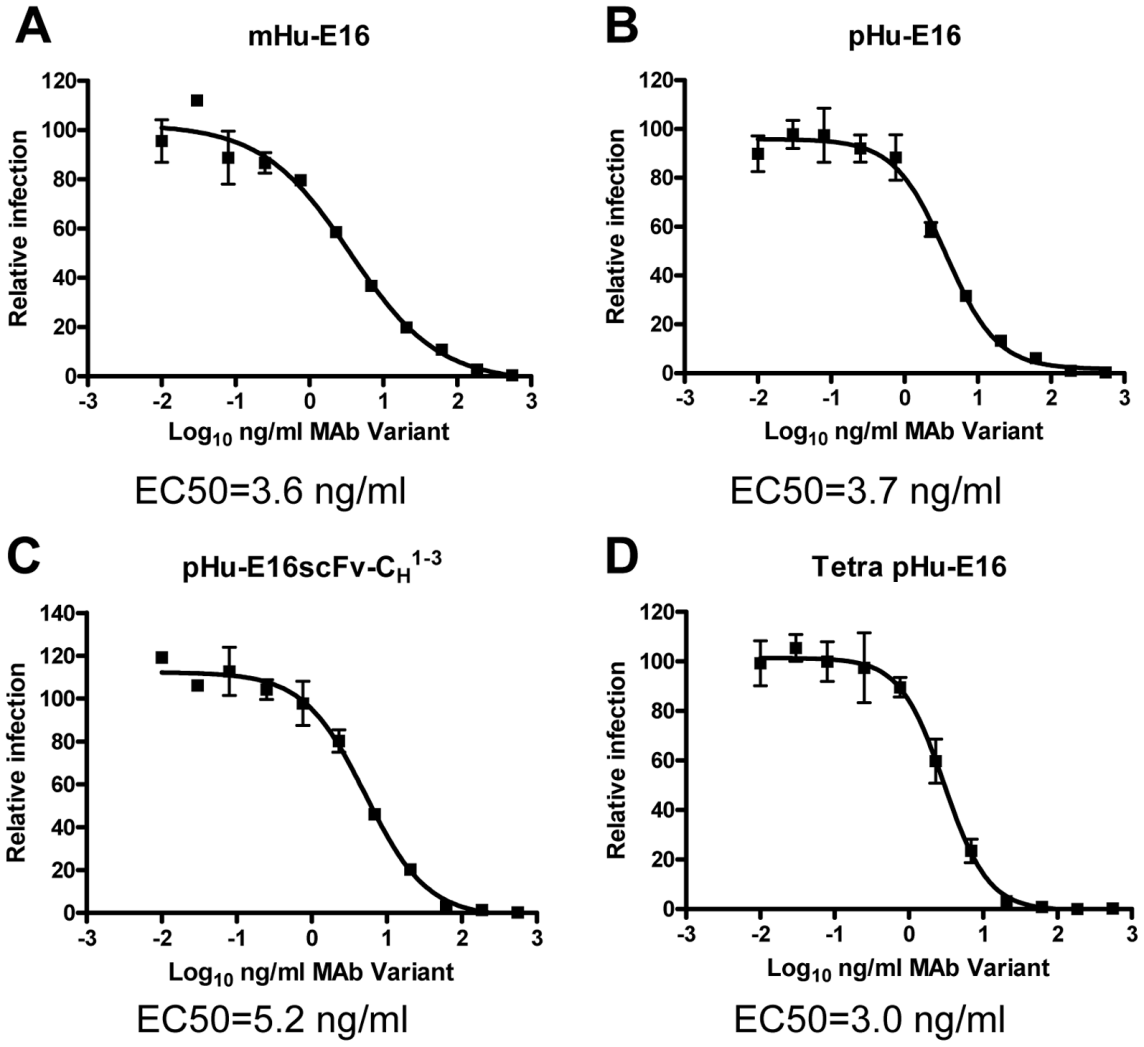
Neutralization of WNV by pHu-E16 variants. WNV was incubated with serial dilutions of mHu-E16 (positive control 1), or pHu-E16 (positive control 2), pHu-E16scFv-C_H_^1-3^, or Tetra pHu-E16 and used to infect Vero cells. Cells were then fixed, permeabilized, analyzed by focus reduction assay and quantitated by Biospot analysis. Mean ± SEM is shown from one of several independent experiments. EC50 values are listed below the graphs.

To evaluate the neutralization potential of pHu-E16 variants, we used a focus reduction neutralization assay that measures antibody inhibition of WNV infection [12], [23]. pHu-E16scFv-C_H_^1-3^ and Tetra pHu-E16 neutralized WNV infection comparably to mHu-E16 and pHu-E16 (**Fig 6**, EC50: mHu-E16 = 3.6 ng/ml, pHu-E16 = 3.7 ng/ml, pHu-E16scFv-C_H_^1-3^ = 5.2 ng/ml, Tetra pHu-E16 = 3.0 ng/ml, respectively). Thus, E16scFv-C_H_^1-3^ and Tetra pHu-E16 retained potent neutralizing activity against infectious WNV.

### pHu-E16 scFv-C_H_^1-3^ and Tetra pHu-E16 have potent prophylactic and therapeutic potential

**Figure 7:**
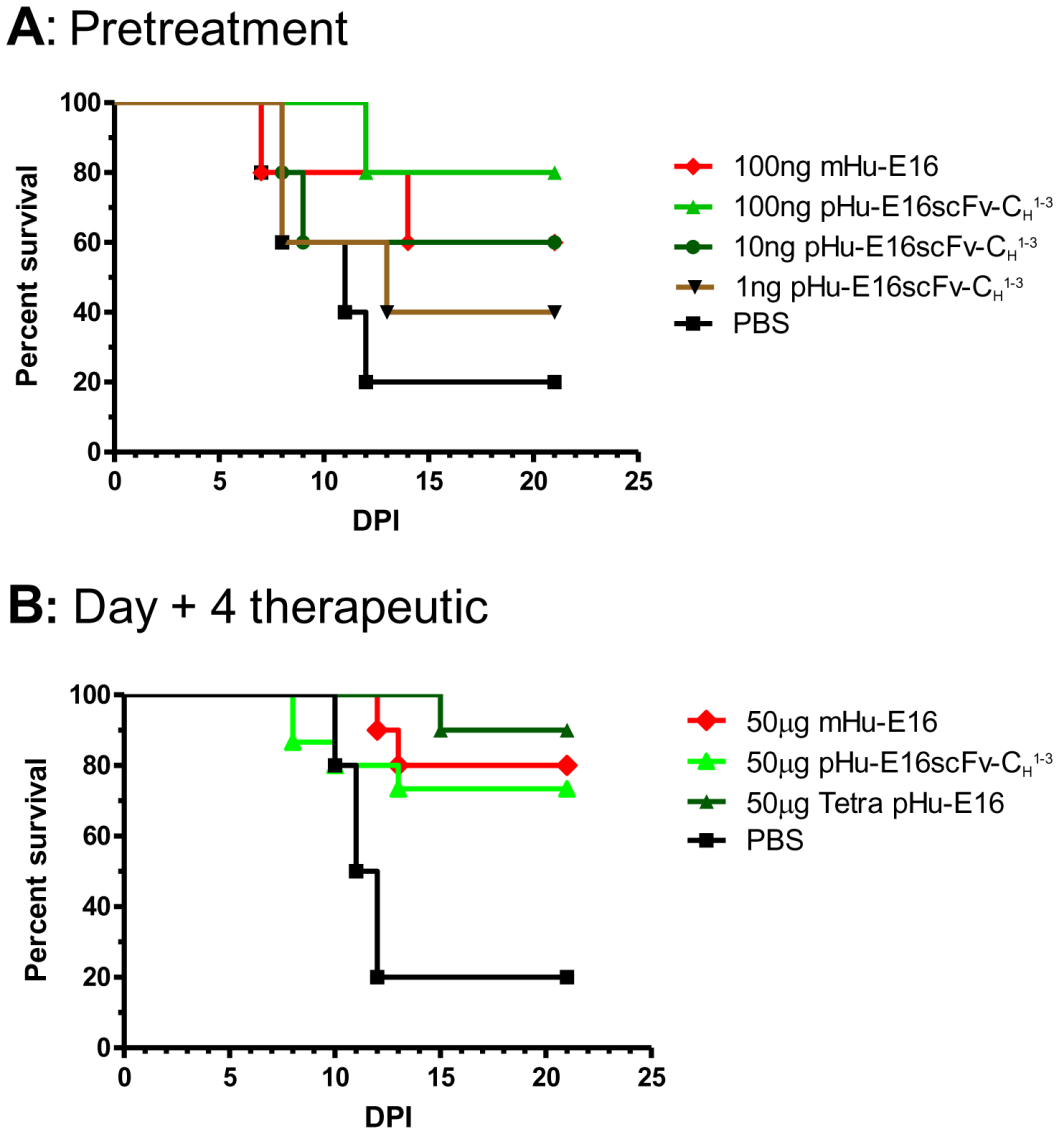
pHu-E16 variants mediated protection in mice. **A**. Five week-old C57BL/6 mice were passively transferred saline, 100 ng of mHu-E16, or serial 10-fold increases in dose (ranging from 1 to 100 ng, *n* = 10 mice per dose) of pHu-E16scFv-C_H_^1-3^ via an intraperitoneal route on the same day as subcutaneous infection with 10^2^ PFU of WNV. Survival data was pooled from at least two independent experiments and analyzed by log-rank test. **B**. Wild type C57BL/6 mice were infected with 10^2^ PFU of WNV and then given a single 50 μg dose of pHu-E16 scFv-C_H_^1-3^, Tetra pHu-E16 or mHu-E16 via an intraperitoneal route at day +4 after infection. Survival data was pooled from at least two independent experiments (*n* = 10 mice per dose) and analyzed by the log-rank test.

Prophylaxis and therapeutic studies were performed to evaluate the activity of pHu-E16 variants *in vivo*. Pre-treatment studies were performed in 5 week-old wild type C57BL/6 mice (*n*≥10, per group) to compare the concentrations of pHu-E16scFv-C_H_^1-3^ and mHu-E16 that prevent WNV infection. Mice were inoculated with 10^2^ PFU of WNV (New York 2000 strain), which causes a baseline mortality of 80 to 90% in this model [24]. Increasing amounts (1 to 100 ng) of pHu-E16scFv-C_H_^1-3^ or 100 ng of mHu-E16 were administered as a single dose on the day of infection. Result showed that 80% of mice were protected from lethal infection when 100 ng of pHu-E16scFv-C_H_^1-3^ was administered (*P*<0.05), whereas the same dose of mHu-E16 protected 60% of mice (**Fig 7A**). A single injection of as low as 10 ng of pHu-E16scFv-C_H_^1-3^ also prevented mortality (**Fig 7A**).

Post-exposure therapeutic treatments were performed by passively administering a single dose of pHu-E16scFv-C_H_^1-3^ or Tetra pHu-E16 after subcutaneous inoculation of 10^2^ PFU of WNV. Since WNV spreads to the brain in mice by day 4 after infection [25], we investigated the efficacy of pHu-E16 variants at this time point. Initially, a 500 μg dose was used as a direct comparison to our prior published results [11], [25]; similar levels of protection were observed between mHu-E16 and pHu-E16scFv-C_H_^1-3^ (data not shown). To define a lower limit of protection, we used a dose of 50 μg (**Fig 7B**). Notably, 70% of mice were protected from lethal infection when 50 μg of pHu-E16scFv-C_H_^1-3^ was given 4 days after WNV inoculation, similar to that observed with mHu-E16 performed in parallel (**Fig 7B, ***P*>0.6). A single administration of 50 μg of Tetra pHu-E16 protected up to 90% of mice from lethal infection, compared with an 80% survival rated achieved by mHu-E16 (**Fig 7B**). Overall, pHu-E16 scFv-C_H_^1-3^ and Tetra pHu-E16 had at least equivalent prophylactic and therapeutic efficacy in mice compared to mHu-E16.

## Discussion

MAb expression initially was attempted in stable transgenic plants [4]. It suffered from low-yield, long lead time to generate and select transgenic plants, and unstable seed banks largely due to the randomness of transgene integration in the plant genome (position effect), the lack of control in transgene copy number, and the shortage of strong promoters to drive transgene expression [3]. This greatly undermined the cost-saving potential of the plant expression systems. The development of transient expression systems based on deconstructed virus vectors has increased the speed and yield of MAb production in plants [13], [26]–[28]. For example, with the MagnICON and geminiviral vectors, MAbs can be obtained within 10 days of vector infiltration into *N. benthamiana* and lettuce plants with yields up to 1 mg MAb/g LFW [11], [12], [29]–[31]. Although MAb derivatives, such as scFv, diabodies, and RIC have been produced in plants [3], [5], [32], the ability of plants to express and assemble larger or more complex MAb-derived molecules such as IgG-like tetravalent MAbs or bifunctional MAbs has not been reported.

Here, we investigated the feasibility of plant cells for producing such an IgG-like tetravalent MAb against WNV based on MAb Hu-E16. Our results showed that two Hu-E16scFvs each genetically fused to the C_H_^1-3^ and C_L_ fragment of IgG can be produced efficiently in *N. benthamiana* leaves. The Hu-E16scFv-C_H_^1-3^ and Hu-E16scFv-C_L_ efficiently assembled into an IgG-like molecule. Our study also demonstrated that the tetravalent pHu-E16 retained antigen-binding and neutralization activity *in vitro*. Most importantly, a single dose of Tetra-pHu-E16 protected mice at least equivalently as mHu-E16 against a lethal WNV infection several days after exposure. To our knowledge, this is the first example in a plant of expression and assembly of a large and complex IgG-like tetravalent functional molecule, although a structurally unrelated secretory IgA (sIgA) molecule assembled from polypeptides of four gene products was produced in stable transgenic plants [4]. Tetra pHu-E16 was expressed rapidly in *N. benthamiana* leaves and reached its highest level of accumulation 9 days after leaf infiltration. While its expression level is slightly lower than that of pHu-E16, this level is within the range feasible for commercial production [33], and likely can be increased by further genetic optimization [3]. Tetra-pHu-E16 and other variants were recovered efficiently to high (>90%) purity with a two-step purification process. In addition to plant host proteins, this process also effectively removed other contaminants including endotoxin, with its levels ranging from 5.2 to 8.6 EU/ml in the purified product (Q. Chen, unpublished data). The rapid accumulation and assembly of Tetra pHu-16 and the availability of a scalable and current Good Manufacturing Practice (cGMP) compliant purification scheme suggest the viability of such molecules as a platform for novel therapeutic candidates.

Another long-term goal of this research is to increase the efficacy of Hu-E16 MAb as a proof-of-principle for antibody treatment of viral infections in the CNS. Even though mHu-E16 [11], [25], pHu-E16 [11], [25], pHu-E16scFv-C_H_^1-3^, and Tetra pHu-E16 showed excellent efficacy, their window of clinical treatment will be limited via peripheral delivery systems. These current MAb-based molecules cannot accumulate to high levels in the brain unless there is widespread inflammation and breakdown of the BBB. Thus, it would be desirable to develop pHu-E16 variants that can cross the BBB more efficiently. One possible strategy is to develop IgG-like bifunctional MAbs that have a similar structure as the Tetra pHu-E16, but are assembled from scFvs that bind to two disparate antigens. For example, one of the two scFvs could bind to a specific receptor (e.g., insulin receptor [34]) on BBB to facilitate its transport into the brain, and the other scFv would retain its binding to and neutralization of WNV in the brain. The successful production and assembly of Tetra pHu-E16 and the demonstration of its therapeutic activity bring us closer to developing such Hu-E16 bifunctional MAbs to prolong the window of its efficacy.

The N-linked glycosylation of MAbs affects its stability, FcγR and C1q binding, pharmacokinetics, effector function and efficacy [17]. N-linked glycosylation occurs as a series of post-translational modification steps initiated in the ER and processed through different compartments of the Golgi complex of the host cell. Altering the structure of a protein or its folding may affect glycan modification and the corresponding cellular localization [35], [36]. However, the impact of structural variations in the native IgG on N-linked glycosylation has not been studied extensively. Here, we first examined the difference in the N-linked glycans of pHu-E16 IgG and its pHu-E16scFv-C_H_^1-3^ variant. Our results revealed that their N-linked glycan profiles were different from one another. pHu-E16 exhibited the expected complex-type N-linked glycans [18], confirming it was fully processed through the endomembrane system and secreted into the apoplast as designed. In contrast, pHu-E16scFv-C_H_^1-3^ predominantly displayed Man8 and Man9-types of oligomannosidic structures, which indicates incomplete glycan processing and suggests its aberrant localization in ER or ER-derived vesicles. A similar observation in glycoforms and aberrant subcellular deposition was reported for Arabidopsis (*Arabidopsis thaliana*) seed-derived 2G12scFV-Fc, a MAb variant recognizing Human immunodeficiency virus (HIV), and to a lesser extend, for HA78scFv-Fc, an anti-hepatitis A virus MAb also produced in Arabidopsis seed [35]. However, in the same study, leaf-derived HA78scFv-Fc displayed the expected complex-type N-glycans, whereas leaf-produced 2G12scFv-Fc exhibited the ER-typical oligomannosidic glycans and was functionally inactive [35]. These observations suggest that beside the polypeptide component of scFv-Fcs, the type of plant tissue (e.g. seed vs. leaf) and plant species also may impact the N-linked glycosylation status of scFv-Fc variants. It should be noted that the structures of 2G12scFv-Fc and HA78scFv-Fc are different from pHu-E16scFv-C_H_^1-3^, as their scFvs were fused to the Fc fragment (hinge-C_H_^2-3^) of IgG and lack the HC constant region 1 domain (C_H_^1^). To investigate if this was a phenomenon unique to plant cells, we compared the N-linked glycan profiles of the equivalent Hu-E16 counterpart variants produced in mammalian cells. Our results indicated that in addition to the predominant complex-type glycans (GnGnF^6^ and AGnF^6^), mHu-E16scFv-C_H_^1-3^ also had a substantial amount of oligomannosidic glycans, mostly M5 and M7, whereas no such glycans were observed in the native IgG version of mHu-E16. This result suggests that at least for Hu-E16, the unusual N-linked glycosylation of scFv-C_H_^1-3^ is not unique to plants but shared by mammalian cells, even though the percentage and the particular species of oligomannosidic glycans varies between the two cell types.

To provide further insight for this N-glycosylation difference, we expressed three additional pHu-E16 variants with various combinations of HC and LC components. We were particularly interested in examining whether LC or the proper pairing of HC and LC impacted the N-linked glycosylation of MAb and its variants. Co-expression of LC with pHu-E16scFv-C_H_^1-3^ increased its level of complex N-linked glycans percentage. In Tetra pHu-E16, the co-expression and pairing of scFv-C_L_ with scFv-C_H_^1-3^ also increased the amount of complex-type N-linked glycans. Furthermore, the pairing of the HC of pHu-E16 with scFv-C_L_ resulted in a predominantly complex-type N-linked glycosylation pattern, similar to seen with the parent pHu-E16. These results support the hypothesis that C_L_ domain of an antibody is essential for its secretion in the cell [36]. It was suggested previously that HC dimers are retained in the ER by the chaperone BiP and are released only upon binding with C_L_ to ensure its proper folding [37]. Our results corroborate this and highlight the importance of LC pairing with HC for the proper processing of antibody N-linked glycosylation. We speculate that in the absence of LC pHu-E16scFv-C_H_^1-3^ was retained in the ER by BiP, which prevented its transport within the endomembrane system and resulted in the observed ER-typical oligomannosidic glycoform. In contrast, co-expression of LC or pHu-E16scFv-C_L_ enabled the C_L_ domain to release pHu-E16scFv-C_H_^1-3^ from BiP and to transport it to the Golgi compartments for further assembly, glycan processing and secretion. While the N-linked glycosylation of the scFv-C_H_^1-3^ and scFv-Fc variant may be regulated by many factors, our results suggest that the lack of C_L_ and its pairing with the C_H_^1^ contributes to the aberrant N-linked glycosylation and intracellular localization of pHu-E16scFv-C_H_^1-3^ and other previously reported scFv-Fcs; the impact of this process may vary depending on the specific scFv-Fc/scFv-C_H_^1-3^ or the cell type in which it was produced.

We used wild-type (WT) *N. benthamiana* plants to express the pHu-E16 variants. While N-linked glycosylation of proteins in plants is similar to that of mammalian cells, WT plants add plant-specific β-1,2-xylose and core α-1,3-fucose residues to complex N-linked glycans and lack terminal β1,4-Gal and N-acetylneuraminic acid (Neu5Ac) residues [38], [39]. Differences in protein glycosylation between WT plant and mammalian cells remain a reservation of plant-expression platforms for human therapies, because they could produce glycoforms that alter efficacy or result in plant-glycan specific immune responses that can accelerate protein clearance or cause potential adverse effects through immune complex formation. To overcome this challenge, glycosylation pathway in *N. benthamiana* and several other plants has been “humanized” through glycoengineering, for example, by genetically suppressing or eliminating enzymes for the biosynthesis of plant-specific glycans and by introducing glycoenzymes from mammalian cells [39]. These efforts have lead to the creation of a portfolio of “humanized” plant lines that produce MAbs that lack plant-specific glycans and produce glycoforms that are essentially mammalian [39]. For example, a double knockout (ΔXF) *N. benthamiana* plant line suppresses the expression of α-1,3-fucosyltransferase and β-1,2-xylosyltransferase and was used for production of an anti-Ebola MAb 13F6 [31], [40]. 13F6 produced in the ΔXF plant line had no plant-specific N-glycans and 90% of 13F6 had the predicted mammalian glycoform GnGn [31]. In contrast, mammalian cell-produced 13F6 had a mixture of 3–5 glycoforms with the most dominant glycoform (G0) ranging from 35–53% [31]. The high glycan homogeneity of plant-made 13F6 and the lack of core fucose increased its protection of mice against a lethal Ebola virus challenge compared to the mammalian cell produced 13F6 [31]. We plan to express pHu-E16 scFv-C_H_^1-3^, Tetra-E16, and other variants in these “humanized” *N. benthamiana* lines to produce highly uniform mammalian glycoforms.

The N-linked glycosylation pattern in the Fc region of an antibody affects its binding to FcγRs and C1q, and consequently alters its effector function such as antibody-dependent cell mediated cytotoxicity (ADCC) and complement-dependent cytotoxicity (CDC) [17]. The highly homogenous and defined nature of N-glycans displayed by plant-derived MAbs provides the opportunity to develop MAb glycoforms that enhance the efficacy through modulating their effector function. For example, plant-produced 13F6 has a higher affinity to FcγRIII (CD16) than its mammalian cell-produced counterpart, and provided better protection against Ebola virus challenge in mice presumably via enhanced ADCC activity [31]. Here, we found that due to their distinct N-glycoforms, pHu-E16, pHu-E16scFv-C_H_^1-3^, and Tetra pHu-E16 exhibited differential binding to C1q and several FcγRs including CD16V^158^, CD16F^158^ CD32A-R^131^ and CD32A-H^131^, CD32B, and CD64. For example, although pHu-E16 showed reduced binding to all FcγRs and C1q, pHu-E16scFv-C_H_^1-3^ demonstrated slightly enhanced binding to the low-affinity (CD16F^158^) isoform of CD16A and CD64, and maintained similar binding to the high-affinity (CD16V^158^) CD16A isoform as mHu-E16. This diversity of binding affinity to C1q and various FcγRs by an individual MAb variant molecule will allow investigation of the impact of glycan moieties on MAb effector function and the development of MAb variants that are best suited for its efficacy or safety depending on the particular clinical application.

In addition to ADCC and CDC, the interaction between Fc and FcγRs can have pathogenic consequences. For example, one of the major impediments towards developing antibody-based therapeutics for certain flavivirus infections (e.g., Dengue virus) is the at least theoretical risk of ADE, which may render anti-flavivirus MAb treated subjects more susceptible to infection. ADE occurs because sub-neutralizing concentrations of antibodies (including therapeutic MAbs) form complexes with the infecting flavivirus that bind to FcγR-bearing cells, resulting in increased virus uptake and infection [19]–[21]. We investigated whether the unique binding of pHu-E16 variants to FcγRs affected ADE. Our results demonstrated that all pHu-E16 variants lost ADE activity on CD32A-expressing K562 cells. Thus, plant complex-type N-glycans or the mammalian form of oligomannosidic glycans carried by plant-produced pHu-E16 variants do not induce the development of ADE efficiently on cells bearing CD32A, consistent with their loss of binding *in vitro* as shown by SPR. In a separate experiment, we observed that ADE also was eliminated for a glycoengineered plant-derived pHu-E16 that carried the mammalian glycoform of GnGn (data not shown). Collectively, these results demonstrate the potential of plant-made MAbs and their variants to be used as therapeutics against ADE-prone viruses. Although it was previously reported that an aglycosylated version of a MAb can eliminate the risk of ADE [41], [42], the complete removal of N-linked glycans and the resulting abolishment of binding to C1q also may compromise the efficacy of a MAb that uses CDC activity. Plant-derived MAb glycolvariants that minimize ADE but retain C1q binding might have better efficacy than their aglycosylated counterparts. While ADE is not a critical issue for WNV infection, it is relevant to the closely related Dengue virus, and some coronaviruses, paramyxoviruses, and lentiviruses [43].

In summary, we established the ability of plant cells to express and assemble a large and complex IgG-like tetravalent MAb variant. The demonstration of *in vitro* and *in vivo* therapeutic function of Tetra pHu-E16 also supports the feasibility of developing bifunctional MAbs that can cross the BBB and extend the window of treatment for WNV infection. This may lead to the generation of better antibody-based therapeutics against WNV and other neurological diseases. This study also highlights the importance of HC and LC pairing for MAb N-glycan processing and intracellular localization, which contributes to our understanding of this basic biological process in plant and animal cells. Finally, our study suggests that structural difference between MAb variants could impact N-linked glycosylation patterns, which in turn can affect *in vivo* efficacy and safety.

## Materials and Methods

### Expression of pHu-E16 MAb variants in *N. benthamiana* leaves

A plasmid containing the pHu-E16 MAb sequence [11] was used as template for the construction of the pHu-E16scFv. Primers H5 (5′ GAATTCACAA TGGGATGG 3′) and H3 (5′ CCTCCTGAAG TTGAACCAGA AGACACAGTA ACAGTAG 3′) were used to amplify the pHu-E16 V_H_, and primers L5 (5′ GGTTCAACTT CAGGAGGAGG ATCAGGTGGT GGTTCAGGAG GTGGAGGATC TTCT GATATC GTTATGACAC AATC 3′) and L3 (5′ TGCTAGCTT TGATCTCCAA CTTAGTTCC 3′) were used to amplify the pHu-E16 V_L_. Subsequently, the V_H_ and V_L_ sequences were linked together by overlapping PCR. The product was cloned using EcoRI and NheI sites into a plasmid, which already contains the coding sequence of the C_H_^1-3^ or C_L_ to yield EcoRI-pHu-E16scFv-C_H_^1-3^-BamHI or EcoRI-pHu-E16scFv-C_L_-BamHI constructs. pHu-E16scFv-C_H_^1-3^ and E16scFv-C_L_ coding sequences were then cloned into plant expression vector pICH11599 and pICH21595, respectively with EcoRI and BamHI restriction enzymes. The construction of pHu-E16 HC and pHu-E16 LC in pICH11599 and pICH21595 has been described previously [11]. None of the pHu-E16 constructs contains the ER-retention signal KDEL. Plant expression vectors were transformed individually into *A. tumefaciens* GV3101 by electroporation as previously described [44]. Wild-type *N. benthamiana* plants were grown and Agroinfiltrated or co-Agroinfiltrated with GV3101 strains containing the pHu-E16scFv-C_H_^1-3^, pHu-E16scFv-C_H_^1-3^/LC, HC/pHu-E16scFv-C_L_ or pHu-E16scFv-C_H_^1-3^/ pHu-E16scFv-C_L_ 3′ modules along with their respective 5′ modules and an integrase construct as described previously to express pHu-E16 variants [11], [45]. To enhance the assembly of pHu-E16scFv-C_H_^1-3^/LC, HC/pHu-E16scFv-C_L_ and Tetra pHu-E16 molecules, the OD_600_ ratio of 4∶1 for HC-derived construct: LC-derived construct was used for *Agrobacterium* co-infiltration.

### Extraction and purification of pHu-E16 variants from plant leaves

For evaluating the temporal pattern of pHu-E16 variant expression, Agroinfiltrated leaves were harvested 5, 6, 7, 8, 9, and 10 dpi. Leaves were harvested 9 dpi for other protein analysis. Extraction and purification of pHu-E16 variants from plant leaves were performed with a method previously reported for pHu-E16 MAb [11]. Briefly, leaves were homogenized in extraction buffer (PBS, 1mM EDTA, 10 mg/ml sodium ascorbate, 10 μg/ml leupeptin, and 0.3 mg/ml PMSF). The crude plant extract was clarified by centrifugation at 17,700×*g* for 30 min at 4°C. pHu-E16 variants in clarified protein extract were purified by a two-step purification process comprised of ammonium sulfate precipitation and protein A affinity chromatography.

### SDS-PAGE, Western blot, ELISA and flow cytometry with yeast surface display

Protein samples were subjected to 10% SDS-PAGE under reducing (5% v/v β-mercaptoethanol) or to 4-20% gradient SDS-PAGE under non-reducing conditions. Gels were stained with Coomassie blue or used to transfer proteins onto PVDF membranes. Horseradish peroxidase (HRP)-conjugated antibodies against human-kappa LC or gamma HC (Southern Biotech) were used for western blot analysis as previously described [11]. pHu-E16 variant expression and antigen binding was examined with an ELISA as described [11]. Briefly, DIII (amino acids 296-415) of the New York 1999 strain of WNV purified from *E. coli* [46], [47] was immobilized on microtiter plates. After incubation with plant protein extract or purified pHu-E16 variants, an HRP-conjugated anti-human-gamma HC antibody (Southern Biotech) was used as the detection antibody. The plates were developed with TMB substrate (KPL Inc). mHu-E16 [25] and pHu-E16 [11] were used as reference standards. Since this ELISA measured the quantity of HC, it provided a molar equivalent of each pHu-E16 variant to pHu-16 rather than their yield in mass. Thus, the yield (μg/g LFW) of each variant was calculated by the formula: (ELISA HC equivalent value)/ (molecular weight of pHu-E16 (150 kDa)) X (molecular weight of the variant) (125 kDa for the pHu-E16scFv-C_H_^1-3^, 170 kDa for pHu-E16scFv-C_H_^1-3^/LC, 175 kDa for HC/pHu-E16scFv-C_L_ and 200 kDa for tetra pHu-E16). Yeast displaying WNV DIII on their surface were generated, stained with MAb variants, and analyzed with a Becton Dickinson FACSCalibur flow cytometer as described [11], [25].

### N-linked glycan analysis

The N-linked glycosylation profile was determined by LC-ESI-MS as previously published [48]. Briefly, purified MAb variants were separated by reducing SDS-PAGE and detected with Coomassie stain, and the HC-containing band was excised from the gel. Upon S-alkylation and tryptic or tryptic/GluC digestion, fragments were eluted from the gel with 50% acetonitrile and separated on a Reversed Phase Column (150×0.32 mm BioBasic-18, Thermo Scientific) with a gradient of 1%–80% acetonitrile. Glycopeptides were analyzed with a quadruple time-of-flight (Q-TOF) Ultima Global mass spectrometer (Waters, Milford, MA, USA). Spectra were summed and deconvoluted for identification of glycoforms. Glycans were annotated according to the ProGlycAn nomenclature (www.proglycan.com).

### Surface plasmon resonance

Binding activity measurements of MAb variants for human C1q and FcγRs (CD16A-158^va l^ (CD16V^158^), CD16A-158^phe^ (CD16F^158^) CD32A-131^arg^ (CD32A-R^131^), CD32A-131^his^ (CD32A-H^131^), CD32B, and CD64) were performed by SPR on a BIAcore 3000 biosensor (GE, Healthcare). WNV E protein was immobilized on the CM-5 sensor chip (∼3000 RU) by an amine coupling kit as recommended by the manufacturer. Hu-E16 variants were bound to the E protein surface at approximately 700RU and normalized to the same level. The capture of Hu-E16 variants on the surface of immobilized antigen prevents the ligand from deactivation and positions Fc regions of it in universal orientation. This is followed by injection of C1q at 50 nmol/L or the soluble monomeric receptors CD16A-158^val^, CD16A-158^phe^ at concentrations of 500 nmol/L, respectively, and flow rate of 30 μl/min for 60 sec with dissociation time 60 sec. Dimeric Fc-G2 (N297Q) fusions of human CD32B, CD32A-R^131^, and CD32A-H^131^, or human soluble CD64 were injected at 200 nmol/L or at 50 nmol/L, respectively. Each receptor was injected in duplicate. Between experiments, the naked antigen surface was regenerated by pulse injection of 10 mM glycine pH 1.5. All binding experiments were performed in 10 mM HEPES, pH 7.4, 150 mM NaCl, 3 mM EDTA, and 0.005% P20 surfactant.

### Antibody-dependent Enhancement Assay

The enhancing activity of the MAbs was determined with CD32A^+^ K562 cells [22] in at least three independent experiments in triplicate using a high-throughput flow cytometry-based assay with GFP pseudo-infectious WNV replicon particles essentially as described [22]. K562 erythroleukemic cells were obtained from the American Type Cell Collection (ATCC CCL-243).

### WNV neutralization

A focus reduction neutralization assay was used to assess the neutralizing activity of pHu-E16 variants against WNV essentially as previously described [23]. Neutralization curves were generated using Prism software to express the percent reduction of spot numbers in samples pre-incubated with mAb compared to wells with virus pre-incubated with medium alone, and EC50 values calculated.

### Efficacy of MAbs in vivo

This study was carried out in strict accordance with the recommendations in the Guide for the Care and Use of Laboratory Animals of the National Institutes of Health. The protocol was approved by the Institutional Animal Care and Use Committee at the Washington University School of Medicine (Assurance Number: A3381-01). Injections were performed under anesthesia that was induced and maintained with ketamine hydrochloride and xylazine, and all efforts were made to minimize suffering. C57BL/6 mice were housed in a pathogen-free mouse facility. Mice received a single dose of purified pHu-E16scFv-C_H_^1-3^, Tetra pHu-E16, or mHu-E16 by intraperitoneal injection the same day or four days after footpad infection with 10^2^ PFU of WNV strain 3000.0259. Kaplan-Meier analysis of survival data was performed using the log-rank test. IC50 analyses were performed by non-linear regression and statistical significances were determined using analysis of variance (ANOVA) and F-tests.
